# Identification of duck liver-expressed antimicrobial peptide 2 and characterization of its bactericidal activity

**DOI:** 10.5713/ajas.18.0571

**Published:** 2018-10-29

**Authors:** Yeojin Hong, Anh Duc Truong, Janggeun Lee, Kyungbaek Lee, Geun-Bae Kim, Kang-Nyeong Heo, Hyun S. Lillehoj, Yeong Ho Hong

**Affiliations:** 1 Department of Animal Science and Technology, Chung-Ang University, Anseong 17546, Korea; 2 Department of Biochemistry and Immunology, National Institute of Veterinary Research, Dong Da, Hanoi 10000, Vietnam; 3 Poultry Research Institute, National Institute of Animal Science, RDA, Pyeongchang 25342, Korea; 4 Animal Biosciences and Biotechnology Laboratory, Agricultural Research Services, United States Department of Agriculture, Beltsville, MD 20705, USA

**Keywords:** Antimicrobial Peptides, Liver-expressed Antimicrobial Peptide 2 (LEAP-2), Duck, Disulfide Bond, Pathogens

## Abstract

**Objective:**

This study was conducted to identify duck liver-expressed antimicrobial peptide 2 (LEAP-2) and demonstrate its antimicrobial activity against various pathogens.

**Methods:**

Tissue samples were collected from 6 to 8-week-old Pekin ducks (*Anas platyrhynchos domesticus*), total RNA was extracted, and cDNA was synthesized. To confirm the duck *LEAP-2* transcript expression levels, quantitative real-time polymerase chain reaction was conducted. Two kinds of peptides (a linear peptide and a disulfide-type peptide) were synthesized to compare the antimicrobial activity. Then, antimicrobial activity assay and fluorescence microscopic analysis were conducted to demonstrate duck LEAP-2 bactericidal activity.

**Results:**

The duck LEAP-2 peptide sequence showed high identity with those of other avian species (>85%), as well as more than 55% of identity with mammalian sequences. *LEAP-2* mRNA was highly expressed in the liver with duodenum next, and then followed by lung, spleen, bursa and jejunum and was the lowest in the muscle. Both of LEAP-2 peptides efficiently killed bacteria, although the disulfide-type LEAP-2 showed more powerful bactericidal activity. Also, gram-positive bacteria was more susceptible to duck LEAP-2 than gram-negative bacteria. Using microscopy, we confirmed that LEAP-2 peptides could kill bacteria by disrupting the bacterial cell envelope.

**Conclusion:**

Duck LEAP-2 showed its antimicrobial activity against both gram-positive and gram-negative bacteria. Disulfide bonds were important for the powerful killing effect by disrupting the bacterial cell envelope. Therefore, duck LEAP-2 can be used for effective antibiotics alternatives.

## INTRODUCTION

With increasing concerns about food and environmental safety with respect to the emergence of antibiotic resistance in pathogens and the increasing presence of antibiotic residues in meat products in the last few decades, novel strategies for alternatives to antibiotics to reduce the usage of antibiotic growth promoters (AGPs) in poultry production are actively under development [1]. In particular, the limited availability of drug alternatives to manage avian diseases poses a major economic challenge in the European Union where AGPs have been banned since 2006.

Antimicrobial peptides (AMPs) are important peptides of innate immune systems. These peptides are composed of 12 to 50 amino acids, which include two or more positively charged residues and a hydrophobic residue [2–4]. In general, AMPs are known to have antimicrobial activity against gram-negative bacteria, gram-positive bacteria, fungi, viruses, parasites, and even cancer cells [5,6]. The general target of AMPs is the bacterial cell membrane, although some AMPs act to inhibit DNA, RNA, and/or protein synthesis. Other properties of AMPs include the induction of cytokine release, cell proliferation, wound healing, and chemotaxis [7,8]. In mammals, AMPs are synthesized primarily by phagocytes or epithelial cells. Conversely, many insect AMPs are produced predominantly by the fat body, the functional equivalent of the liver, and secreted into the hemolymph after septic injury [9–11].

There are various infectious diseases that poultry can take. Avian influenza, avian tuberculosis, fowl cholera, fowl pox, infectious bronchitis, infectious bursal disease, Marek’s disease, mycoplasmosis, necrotic enteritis and salmonellosis, etc. are triggered by viruses, parasites and bacteria. The AMPs can be used for poultry infectious disease control because it have antiviral, anti-parasite and antibacterial activities [5,6].

Liver-expressed antimicrobial peptide 2 (LEAP-2) was first isolated from a human in 2003 [12] and has been reported from other species, including mice, cattle, pigs, and chicken [13]. In most species, LEAP-2 is a cysteine-rich and cationic protein [14,15], which generally has a conserved core structure with two disulfide bonds that play a crucial role in bacterial killing [16]. In poultry, LEAP-2 sequences have been characterized in chicken and Japanese quail [17,18], but duck LEAP-2 has not yet been characterized.

Therefore, in this study, we identified and characterized the duck *LEAP-2* gene to determine its antimicrobial activity against gram-positive and gram-negative bacteria.

## MATERIALS AND METHODS

### Tissue collection

Tissue samples, including the muscle, kidney, thymus, lung, spleen, liver, bursa of Fabricius, duodenum, jejunum, caeca, and cloaca, were collected from 6 to 8-week-old Pekin duck (*Anas platyrhynchos domesticus*) at a local farm of Anseong city, Republic of Korea. The samples were washed with phosphate-buffered saline (PBS, pH 7.4), frozen immediately in liquid nitrogen, and stored at −80°C for future use. The protocol for this experiment was approved by the Institutional Animal Care and Use Committee at Chung-Ang University.

### RNA extraction and cDNA synthesis

The total RNA of tissue samples was extracted with TRIzol Reagent (Invitrogen, Carlsbad, CA, USA) according to the manufacturer’s protocol. For cDNA synthesis, 2 μg of RNA samples were treated with DNase I (Thermo Scientific, Waltham, MA, USA) and incubated for 30 min at 37°C and then desalted using ethylenediaminetetraacetic acid (Thermo Scientific, USA). Reverse transcription was performed using the RevertAid First Strand cDNA Synthesis Kit (Thermo Scientific, USA) according to the manufacturer’s protocols.

### Quantitative real-time polymerase chain reaction

To analyze the transcripts of duck *LEAP-2* in various organs, the following primers were designed using Primer-BLAST (https://www.ncbi.nlm.nih.gov/tools/primer-blast/): glyceraldehyde-3-phosphate dehydrogenase (*GAPDH*) forward 5′-TGG TGCTGATACGTTGTGGAGTC-3′ and reverse 5′-AGC TGAGGGGGCGGAGATGA-3′, duck-LEAP-2 forward 5′-TG ACACCGTTCTGGAGAGGA-3′ and reverse 5′-GATCTG AGGAAGCAGCGGTT-3′. Quantitative real-time polymerase chain reaction (qRT-PCR) was performed using AMPIGENE qPCR Green Mix Lo-ROX (Enzo Life Sciences, Lausen, Switzerland) according to the manufacturer’s instructions, using the LightCycler 96 system (Roche, Indianapolis, IN, USA). The duck *GAPDH* gene was used as a control to normalize for RNA quantity. The relative quantification of gene-specific expression was calculated using the 2^−ΔΔCt^ method following normalization with the *GAPDH* gene expression level [19].

### Cloning of duck liver-expressed antimicrobial peptide 2

The primers were designed using DNASTAR (DNASTAR Incorporation, Madison, WI, USA) for amplification of the *LEAP-2* open reading frame from the predicted duck *LEAP-2* cDNA sequence (ENSAPLT00000011688.1). The PCR product was amplified using the specific primers forward 5′-CG GGATCCATGCACTCTTTGAAAGTCATGGC-3′ and reverse 5′-CGGAATTCCTCGGAGGCGGATCTGAG-3′ (BamHI and EcoRI restriction enzyme sites are underlined) with a DreamTaq Green PCR Master Mix (2×) (Thermo Scientific, USA). The PCR amplification was achieved under the following condition: a pre-denaturation step at 95°C for 5 min, a denaturing step at 94°C for 45 s, an annealing step at 55°C for 45 s, an extension step at 72°C for 45 s for 35 cycles, and a final extension at 72°C for 5 min. The PCR products were purified using the PureLink Quick Gel Extraction Kit (Invitrogen, USA), cloned into the pCR2.1-TOPO vector (Invitrogen, USA), and transformed using *Escherichia coli* (*E. coli*) TOP 10 competent cells (Invitrogen, USA) according to the manufacturer’s protocol. Through blue-white screening, the positive clones were picked out and then cultured overnight in Luria-Bertani (LB) broth (with 50 μg/mL ampicillin). Plasmids were extracted using NucleoSpin Plasmid (Macherey-Nagel, Düren, North Rhine-Westphalia, Germany) and sequenced at Genotech (Daejeon, Korea).

### Recombinant protein expression and purification

The duck LEAP-2/pCR2.1-TOPO vector was digested with the restriction enzymes EcoRI and BamHI (Promega, Madison, WI, USA). The protein expression vector pET32a (Novagen, Madison, WI, USA) was also digested with the same restriction enzymes. The digested fragments were purified from the agarose gel using the PureLink Quick Gel Extraction Kit (Invitrogen, USA) and were ligated using T4 DNA Ligase (Invitrogen, USA). The ligated vector and insert were transformed into One Shot BL21 (DE3) Chemically Competent *E. coli* (Invitrogen, USA) and sequenced. Positive clones were incubated at 37°C overnight on a shaking incubator at 225 rpm in LB broth with ampicillin (50 μg/mL). The bacteria culture was then induced for recombinant protein expression with 1 mM isopropyl-β-D-thiogalctopyranoside (USB Corporation, Cleveland, OH, USA) for 4 h at 28°C, and the bacteria were centrifuged at 5,000×*g* for 15 min. The duck LEAP-2 recombinant protein was extracted with B-PER Bacterial Protein Extraction Reagent (Thermo Scientific, USA) and purified using HisPur Cobalt Resin (Thermo Scientific, USA). Recombinant duck LEAP-2 was eluted using 250 mM imidazole and analyzed by sodium dodecyl sulfate-polyacrylamide gel electrophoresis and western blotting using 6× His-tag antibody (Thermo Scientific, USA).

### Peptide synthesis

The mature peptide of duck LEAP-2 was synthesized and purified to a >90% level using high-performance liquid chromatography by GL Biochem Ltd. (Shanghai, China). Two kinds of duck LEAP-2 peptides were synthesized about this mature peptide sequence. MTPFWRGVSLRPIGASCRDNSECITML CRKNRCFLRSASE; the one is linear type and the other peptide have two disulfide bonds (C17–C28, C23–C33).

### Pathogenic bacteria

The bacterial species used in this experiment included two gram-positive bacteria strains, *Listeria monocytogenes* (*L. monocytogenes*) ATCC 19115 and *Staphylococcus aureus* (*S. aureus*) ATCC 27664, and four gram-negative bacteria strains, *E. coli* ATCC 43888, *Salmonella enterica* serovar Enteritidis YHS 383, *Salmonella enterica* serovar Choleraesuis YHS 386, and *Salmonella enterica* serovar Typhimurium ATCC 43174.

### Antimicrobial activity assay

Bacteria were cultured overnight at 37°C in LB broth for *E. coli*, and in tryptic soy broth for the other bacteria, and suspended to 5.0×10^5^ colony-forming units (CFU)/mL in PBS (pH 7.4). To measure antimicrobial activity, 25 μL of bacteria were added into 96-well microtiter plates and diluted duck LEAP-2 peptides were dispensed to final concentrations of 50, 100, 150, and 200 μg/mL. After 3 h of incubation at 37°C, surviving bacteria were counted using a standard colony counting assay according to the following formula: cell survival % = (treatment CFU/negative control CFU)×100. To determine the killing kinetics, *L. monocytogenes* and *S. aureus* were cultured overnight in tryptic soy broth and suspended to 5.0×10^5^ CFU/mL in PBS (pH 7.4). Bacteria were added into 96-well microtiter plates with a final peptide concentration of 200 μg/mL. The bacteria and peptide mixture was incubated for 0, 30, 60, 90, and 180 min at 37°C. Surviving bacteria were counted using a standard colony counting assay.

### Fluorescence microscopic analysis

*E. coli*, *S. aureus*, and *L. monocytogenes* (5.0×10^5^ CFU/mL) in PBS were incubated with a 200 μg/mL (final concentration) of the disulfide-LEAP-2 peptide for 3 h at 37°C. After incubation, the cells were washed with PBS and stained with LIVE/DEAD BacLight Bacterial Viability Kits (Invitrogen, USA) according to the manufacturer’s instructions. In brief, the bacteria were incubated for 15 min with SYTO9 green fluorescent protein and with propidium iodide in a dark room. The cells were then mounted onto glass slides and examined using EVOS FLoid Cell Imaging Station (Invitrogen, USA).

### Bioinformatics analysis

Purified plasmids were sequenced at Genotech (Korea). To compare the cloned duck *LEAP-2* sequence with sequences in GenBank, the data were analyzed using a Nucleotide Basic Local Alignment Search Tool (nBLAST) search (http://www.ncbi.nlm.nih.gov/BLAST/). Protein identification was performed using the Expert Protein Analysis System (https://www.expasy.org/) for determination of the molecular weight and theoretical isoelectric point (pI). Amino acid multiple alignments were generated using CLUSTALW (http://www.genome.jp/tools/clustalw/) and the MEGA 7 program. The protein structure was predicted by RaptorX (http://raptorx.uchicago.edu/) and FirstGlance in Jmol (http://www.bioinformatics.org/firstglance/fgij/).

### Statistical analysis

The qRT-PCR data were analyzed by one-way analysis of variance followed by Duncan’s multiple comparison test using IBM SPSS Statistics 23 software (IBM SPSS Statistics 23 for Windows, Chicago, IL, USA) and antimicrobial activities were analyzed by one-way analysis of variance followed by Dunnett’s multiple comparison test. The antimicrobial activity of disulfide and linear LEAP-2 was analyzed by the student’s t-test using IBM SPSS Statistics 23 software. The data were expressed as means±standard error of the mean and differences were considered statistically significant at p<0.05.

## RESULTS

### Bioinformatics analysis

**Figure 1:**
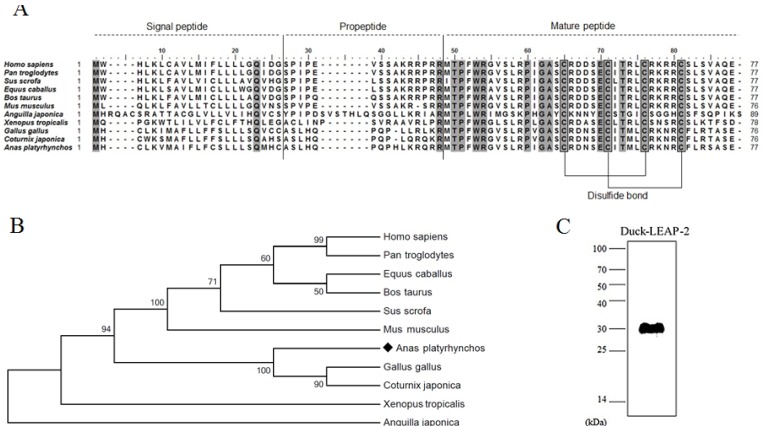
(A) Multiple alignment of the duck LEAP-2 amino acid sequence with several vertebrate LEAP-2 homologs. Conserved sequences are highlighted in gray boxes. (B) Phylogenetic analysis of the complete amino acid sequence of LEAP-2 using the MEGA7.0 program. The values indicate the percentage of trees in which this grouping occurred after bootstrapping. (C) Western blot of recombinant duck LEAP-2 using anti-6× histidine antibody. LEAP-2, liver-expressed antimicrobial peptide 2.

**Figure 2:**
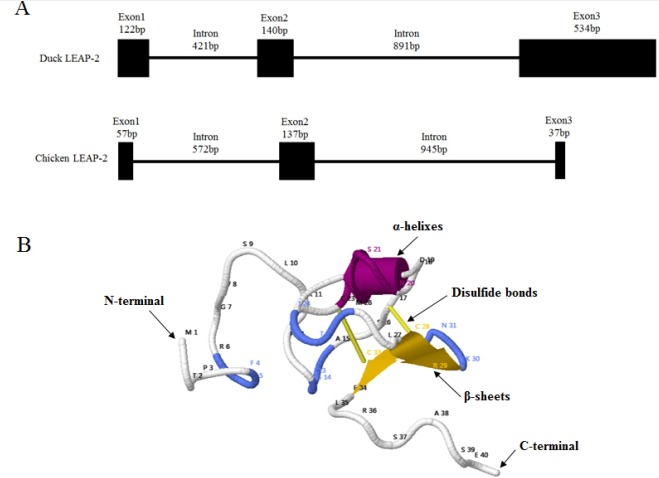
(A) Comparison of the exon-intron compositions of the duck *LEAP-2* and chicken *LEAP-2* gene structures. The black rectangles indicate exons and introns in line. (B) Structural analysis of duck LEAP-2 protein. The letters on the line are the amino acid abbreviations, and the numbers next to the abbreviations indicate the amino acid positions in the protein. Alpha helices are shown as red “rockets”, beta strands are shown as yellow planks, blue lines indicate turns, and yellow sticks indicate disulfide bonds. *LEAP-2*, liver-expressed antimicrobial peptide 2.

The duck LEAP-2 peptide was aligned with other avian, fish, amphibian, and mammalian LEAP-2 sequences (Figure 1A). The duck LEAP-2 peptide sequence showed high identity with those of other avian species (>85%), as well as more than 55% of identity with mammalian sequences, 32% identity with eel, and 46% identity with frog (Table 1); overall, there was more than 60% and 89% similarity with LEAP-2 of mammalian and avian species, respectively. However, the mature peptide sequence showed more than 90% similarity with those of other avian, mammalian, and amphibian species. In addition, the mature peptide showed four conserved cysteine residues that consist of disulfide bonds. LEAP-2 sequences could be clearly divided into two groups, with avian species in one group and mammals in the other group. In phylogenetic tree analysis, duck LEAP-2 showed high similarity with chicken LEAP-2 (Figure 1B, Table 1). Therefore, we compared the chicken and duck LEAP-2 gene structures with respect to the exon-intron organization (Figure 2A). Both the duck and chicken *LEAP-2* genes were composed of 3 exons and 2 introns; however, the number of base pairs of the exons and introns differed. We also performed structure analysis of duck LEAP-2 protein, and confirmed that duck LEAP-2 is composed of one alpha-helix, two beta-sheets, and two disulfide bonds (Figure 2B).

### Production of recombinant duck liver-expressed antimicrobial peptide 2 protein

We produced recombinant LEAP-2 protein using an *E. coli* expression system. The molecular weight of duck LEAP-2 was determined to be 8.9 kDa (Signal peptide-propeptide-mature peptide). Because the fusion protein molecular weight in pET32a is 21 kDa, the final molecular weight of LEAP-2 was approximately 30 kDa. We also confirmed the expression of recombinant LEAP-2 by western blotting using 6× His-tag antibody (Figure 1C).

### Tissue expression profiles of duck liver-expressed antimicrobial peptide 2

**Figure 3:**
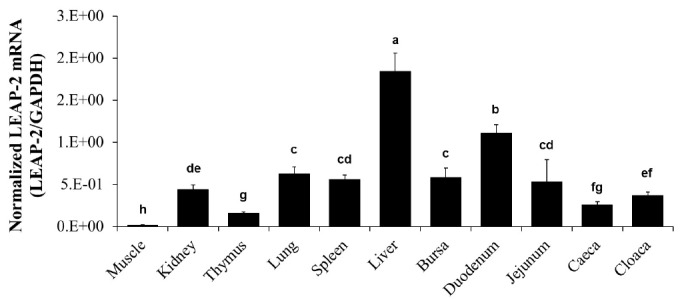
Duck *LEAP-2* mRNA expression levels in various organs. Duck *LEAP-2* mRNA transcript levels were determined in the muscle, kidney, thymus, lung, spleen, liver, bursa of Fabricius, duodenum, jejunum, caeca, and cloaca by real-time polymerase chain reaction. The data are presented as normalized mRNA levels to *GAPDH*. *LEAP-2*, liver-expressed antimicrobial peptide 2; *GAPDH*, glyceraldehyde-3-phosphate dehydrogenase.

We measured the mRNA level of duck *LEAP-2* in the muscle, kidney, thymus, lung, spleen, liver, bursa of Fabricius, duodenum, jejunum, caeca, and cloaca by real-time PCR. The *LEAP-2* mRNA transcript level was the highest in the liver with duodenum next, and then followed by lung, spleen, bursa and jejunum and was the lowest in the muscle (Figure 3).

### Antimicrobial activity of synthetic duck liver-expressed antimicrobial peptide 2 peptide

**Figure 4:**
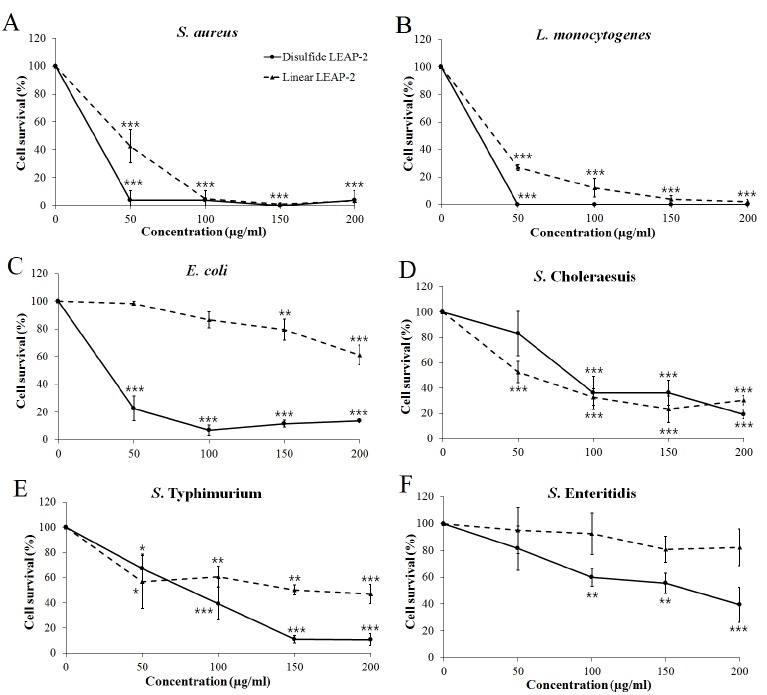
Antibacterial activity of synthetic duck LEAP-2 peptides. The pathogens were treated with linear LEAP-2 and disulfide LEAP-2 peptides and incubated for 3 h at 37°C. (A) *Staphylococcus aureus* ATCC 276674, (B) *Listeria monocytogenes* ATCC 19115, (C) *Escherichia coli* ATCC 43888, (D) *Salmonella enterica* serovar Choleraesuis YHS 386, (E) *Salmonella enterica* serovar Typhimurium ATCC 43174, (F) *Salmonella enterica* serovar Enteritidis YHS 383. The solid line is the disulfide LEAP-2 peptide and the dotted line is the linear LEAP-2 peptide. Cell survival (%) was calculated as (treatment CFU/negative control CFU)×100. LEAP-2, liver-expressed antimicrobial peptide 2; CFU, colony-forming units. Each data point is the mean±standard error of the mean (* p<0.05, ** p<0.01, and *** p<0.001).

We synthesized two peptides, a disulfide bond LEAP-2 and linear LEAP-2 peptide, to measure the LEAP-2 antimicrobial activity. We treated the peptides with gram positive and gram negative bacteria for 3 hours by diversifying the concentration. LEAP-2 showed a significant killing effect for the gram positive bacteria *S. aureus* and *L. monocytogenes* (Figure 4A, 4B). In particular, the disulfide LEAP-2 peptide killed most of the *S. aureus* and *L. monocytogenes* at 50 μg/mL. Gram-negative bacteria were also killed by LEAP-2, but the efficiency was lower than that against gram-positive bacteria (Figure 4C–4F). As shown in Figure 4C, linear LEAP-2 killed 60% of *E. coli* at 200 μg/mL. However, disulfide LEAP-2 killed more than 90% of *E. coli* at 50 μg/mL. The two peptides showed similar patterns against *S*. Choleraesuis, in which more than 70% of the cells were inhibited at 200 μg/mL. Linear LEAP-2 showed a 50% killing effect for *S*. Typhimurium, whereas disulfide LEAP-2 inhibited 90% of the cell proliferation at 200 μg/mL. *S*. Enteritidis was also inhibited by 60% with the disulfide LEAP-2, although it was not inhibited by linear LEAP-2. Taken together, LEAP-2 showed more powerful antibacterial activity to gram positive bacteria than gram negative bacteria. Also, disulfide LEAP-2 showed better killing effect than linear LEAP-2 peptide by showing a stronger bactericidal effect except for *S*. Cholerasuis.

### Killing kinetics of synthetic duck liver-expressed antimicrobial peptide 2

**Figure 5:**
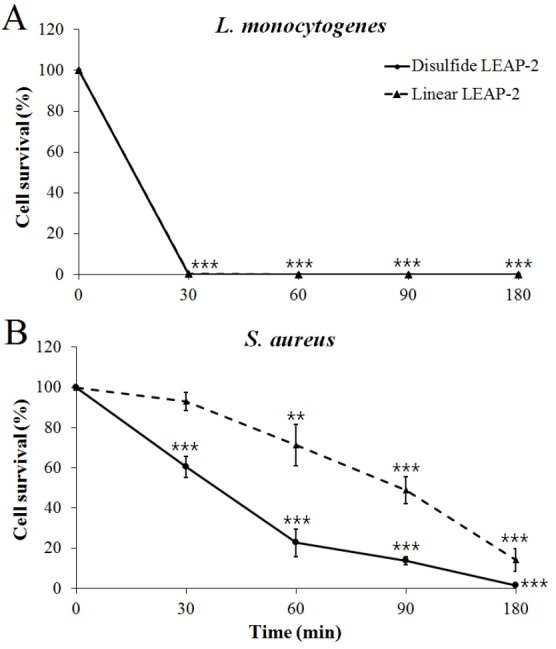
Killing kinetics of synthetic duck LEAP-2 against gram-positive bacteria. (A) *Listeria monocytogenes* ATCC 19115, (B) *Staphylococcus aureus* ATCC 276674. The pathogens were treated with linear LEAP-2 and disulfide LEAP-2 and incubated for 30, 60, 90, and 180 min at 37°C. The solid line is the disulfide LEAP-2 peptide and the dotted line is linear LEAP-2 peptide. Cell survival (%) was calculated as (treatment CFU/negative control CFU)×100. LEAP-2, liver-expressed antimicrobial peptide 2; CFU, colony-forming units. Each data point is the mean±standard error of the mean (* p<0.05, ** p<0.01, and *** p<0.001).

*L. monocytogenes* and *S. aureus* were used to determine the killing kinetics of duck LEAP-2 over time. Both the disulfide LEAP-2 and linear LEAP-2 peptides effectively killed all of the *L. monocytogenes* in only 30 min (Figure 5A). In addition, both disulfide LEAP-2 and linear LEAP-2 killed *S. aureus* gradually over time, with disulfide LEAP-2 showing a much better effect (Figure 5B).

### Fluorescence microscopic analysis for live/dead pathogens

**Figure 6:**
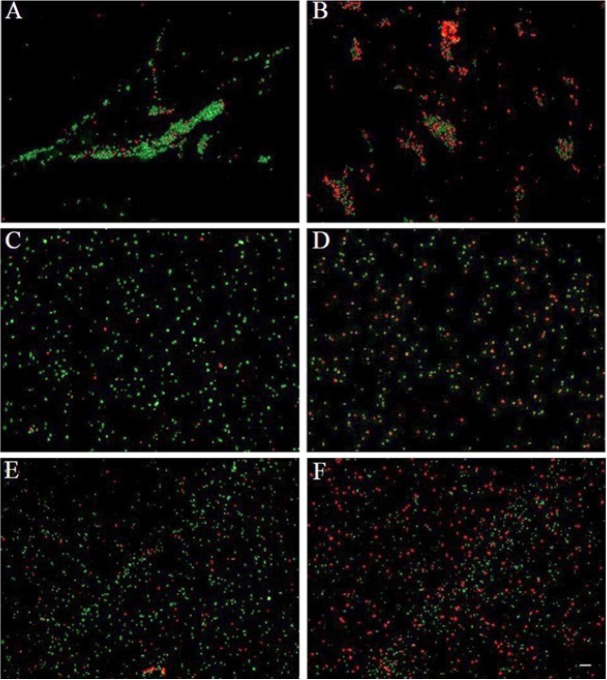
Fluorescence microscopy analysis of disrupted bacteria. Bacteria were treated with disulfide liver-expressed antimicrobial peptide 2 peptide (200 μg/mL) and incubated for 3 h at 37°C. Bacteria were stained with a LIVE/DEAD BacLight Bacterial Viability Kit. (A) Control *Escherichia coli*, (B) treated *Escherichia coli*, (C) control *Staphylococcus aureus*, (D) treated *Staphylococcus aureus*, (E) control *Listeria monocytogenes*, (F) treated *Listeria monocytogenes*. Green fluorescence indicates live bacteria and red fluorescence indicates dead bacteria with damaged membranes. Scale bar = 7 μm.

To visualize the killing effect of duck LEAP-2, a live/dead staining method was carried out. *L. monocytogenes*, *S. aureus*, and *E. coli* were incubated with the disulfide peptide for 3 h before staining. Many of the cells in the peptide treatment group showed red fluorescence, indicating that most of the cells were dead (Figure 6), whereas the cells of the control group (no peptide treatment) showed only green fluorescence, indicating that most of the cells were alive.

## DISCUSSION

AMPs of animals are potentially powerful antibiotics substitutes. LEAP-2 is a generally well-known AMPs in mammals, avian, and fish that has bactericidal effects [12,15,17,18,20].

In general, AMPs have to bind to the bacterial cell membrane to exert their effects [6]. Because most bacterial cell membranes have a net negative charge due to the presence of anionic phospholipids, lipopolysaccharide on the surface of gram-negative bacteria, and teichoic acids on the surface of gram-positive bacteria, the cationic AMPs can bind to the cell membrane. After binding to the cell surface, AMPs create a pore such as a toroidal pore, carpet, or barrel stave [21], which then ruptures the bacterial cell to ultimately kill the bacteria [22].

In the current study, we first identified duck LEAP-2 and characterized its antimicrobial function against pathogens. In addition, we predicted the duck *LEAP-2* gene structure as a signal peptide, pro-peptide, and mature peptide based on the LEAP-2 sequences of mammals and other avian species (Figure 1). Duck LEAP-2 shows conserved mature peptide sequences and two disulfide bonds similar to those of other species based on alignment of the protein sequence and phylogenetic analysis. In general, disulfide bonds play an important role in the folding and stability of certain proteins [23–26]. A previous study also showed that conserved disulfide bridges in avian-β-defensin-12 are essential for the chemotactic property and maximum antimicrobial activity [27]. Therefore, we could predict that these disulfide bonds are an important component for the bactericidal effect of duck LEAP-2.

In mammals, human, pig, and horse, *LEAP-2* mRNA has been shown to be expressed mainly in the liver, kidney, and intestine [12,14,28,29]. In addition, chicken *LEAP-2* mRNA was reported to be mainly expressed in the liver, kidney, small intestine, and lung [18,30,31]. As expected, we found that the duck *LEAP-2* expression levels were the highest in the liver, with the remaining distribution corresponding to some extent with that of the chicken (Figure 3).

To confirm the importance of the disulfide bond in the bactericidal effect, we synthesized two kinds of peptides, linear and disulfide. The disulfide LEAP-2 peptide showed a more powerful bactericidal effect than the linear form except against *S*. Choleraesuis. The AMP should have contact with bacterial cell membrane to show antibacterial activity. So, the peptide structure will be important for affinity. Originally, duck LEAP-2 has two disulfide bonds in mature peptide, so we think that original structure of LEAP-2 has more potent killing effect. Considering the mode of action of most AMPs, the conserved disulfide bonds in LEAP-2 likely play a crucial role in the stability of the peptide.

We tested the duck LEAP-2 recombinant protein against various bacterial strains (data not shown). Unfortunately, we did not observe a substantial bacterial killing effect. Prokaryotic protein expression differs from the eukaryotic protein expression system, and due to translational modifications such as protein glycosylation, some recombinant proteins expressed in prokaryotes might not be suitable for functional recombinant protein production [32]. Therefore, we suggest that the large size of the fusion protein affected the LEAP-2 structure and function [33].

With live/dead staining, we demonstrated that the bacteria cell membranes were disrupted by LEAP-2 peptides (Figure 6). SYTO9 green fluorescent protein stains the nucleic acids of all bacteria with intact membranes, whereas propidium iodide can only penetrate bacteria cells with damaged membranes, causing a reduction in the SYTO9 fluorescence when both dyes are present. Therefore, we could confirm that the mode of action of LEAP-2 is membrane destruction. The main mechanism for membrane destruction is the binding of AMPs to the bacterial cell envelope. As mentioned above, lipoteichoic acid and lipopolysaccharide confer the bacterial cell envelope with a negative charge, and most AMPs have a positive charge [2–4]; thus, this mode of action suggests that LEAP-2 also has an overall positive charge and results in cell envelope disruption.

The net charge of a protein is affected by its own pI and the pH of the surrounding environment. When present at a pH lower than their pI, proteins obtain a net positive charge. In contrast, when present at a pH above their pI, they obtain a net negative charge [34]. The pI of the mature duck LEAP-2 peptide was determined to be 9.11. Because the physiological pH such as that in the gastrointestinal tract and blood is lower than 9.11, duck LEAP-2 would carry a net positive charge in a physiological environment. Therefore, cationic LEAP-2 would bind to the negatively charged residues of the bacterial cell wall or membrane. After LEAP-2 binds to the bacteria, it will disrupt the cell envelope by making pores, and consequently cause the leakage of cell contents and cell death.

Taken together, our study identified duck LEAP-2 and demonstrated its antimicrobial activity against both gram-positive and gram-negative bacteria. In particular, we identified the importance of the disulfide bond for a powerful killing effect by disrupting the bacterial cell envelope. Therefore, duck LEAP-2 may be used as an effective AMPs to substitute for antibiotics and as a novel disease control agent in the future.

## Figures and Tables

**Table 1:** Similarity (upper diagonal) and identity (lower diagonal) of the duck LEAP-2 amino acid sequence with known LEAP-2 sequences of other species (%)

	Human	Chimpanzee	Pig	Horse	Cow	Mouse	Eel	Frog	Chicken	Quail	Duck	GenBank Acc No.
Human	-	100	89.61	94.8	92.2	88.15	46.75	61.03	64.47	60.52	62.33	NP_443203.1
Chimpanzee	100	-	89.61	94.8	92.2	88.15	46.75	61.03	64.47	60.52	62.33	XP_001164115
Pig	85.71	85.71	-	92.2	90.9	86.84	45.45	57.14	60.52	57.89	62.33	NP_998953.1
Horse	92.2	92.2	90.9	-	93.5	86.84	46.75	59.74	60.52	56.57	61.03	XP_003362867.1
Cow	90.9	90.9	89.61	93.5	-	85.52	45.45	59.74	63.15	59.21	63.63	NP_776984.1.
Mouse	85.52	85.52	81.57	84.21	84.21	-	47.36	56.57	63.15	60.52	64.47	NP_694709.1
Eel	33.76	33.76	33.76	35.06	35.06	35.52	-	42.3	44.73	43.42	41.55	ALB07167.1
Frog	49.35	49.35	48.05	46.75	46.75	46.05	29.48	-	63.15	59.21	57.14	AAI55466.1
Chicken	57.89	57.89	52.63	55.26	57.89	57.89	34.21	51.31	-	90.78	90.78	AAS99322.1
Quail	56.57	56.57	51.31	52.63	55.26	56.57	32.89	50	90.78	-	89.47	BAU36332.1
Duck	57.14	57.14	54.54	55.84	58.44	59.21	32.46	46.75	85.52	85.52	-	ENSAPLP00000010969.1

LEAP-2, liver-expressed antimicrobial peptide 2.
